# Supplementary material for: Elucidating Design Principles for Engineering Cell‐Derived Vesicles to Inhibit SARS‐CoV‐2 Infection
Source: Small. 2022 Apr 7;18(19):2200125. doi: 10.1002/smll.202200125 (PMC9106922; doi:10.1002/smll.202200125)
Supplement: Supplementary file 1 — Supporting Information [file SMLL-18-0-s001.pdf]

NANO small MICRO

Supporting Information

for *Small,* DOI: 10.1002/smll.202200125

Elucidating Design Principles for Engineering Cell-Derived Vesicles to Inhibit SARS-CoV-2 Infection

Taylor F. Gunnels, Devin M. Stranford, Roxana E. Mitrut, Neha P. Kamat, and Joshua N. Leonard**

Supporting Information

Elucidating Design Principles for Engineering Cell-Derived Vesicles to Inhibit SARS-CoV-2 Infection

Taylor F. Gunnels, Devin M. Stranford, Roxana E. Mitrut, Neha P. Kamat, Joshua N. Leonard**

Contents:

- Figures S1-S10
- Tables S1-S11

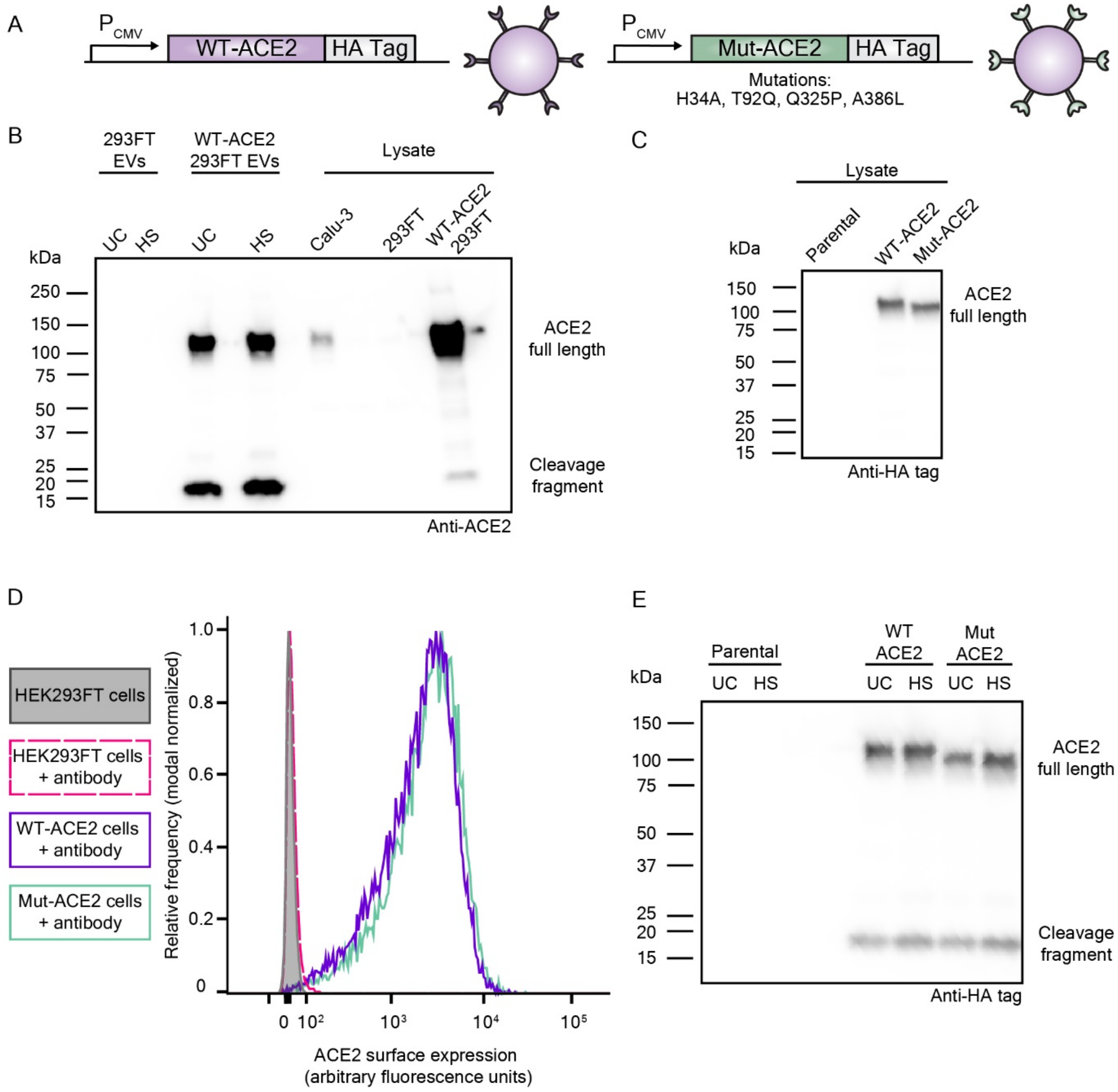

Figure S1. Engineering and characterization of ACE2-expressing cells and ACE2-containing EVs. A) Schematic depicting the ACE2 DNA constructs used in the study and a cartoon of how the constructs are displayed on vesicles. Both WT-ACE2 and Mut-ACE2 are codon optimized, downstream of a cytomegalovirus (CMV) promoter, and fused to a C-terminal HA tag. Mut-ACE2's specific amino acid mutations are identified below the DNA cartoon. B) Western blot using an α-ACE2 primary antibody (Ab15348) to detect ACE2 in EVs and cell lysates in non-engineered HEK293FTs and engineered HEK293FTs. Calu-3s are known to express ACE2 (n = 1). C) Western blot using an α-HA-tag primary antibody to detect transgenic ACE2 in engineered

cell lysates (*n* = 1). D) Flow cytometry analysis of surface staining of parental or engineered cells with a fluorescent, α-ACE2 primary antibody (*n* = 1). E) Western blot using an α-HA-tag primary antibody to detect transgenic ACE2 in engineered EVs (*n* = 2). Within each western blot, equal numbers of particles (for EVs) or amount of protein (for lysate) were loaded in each well.

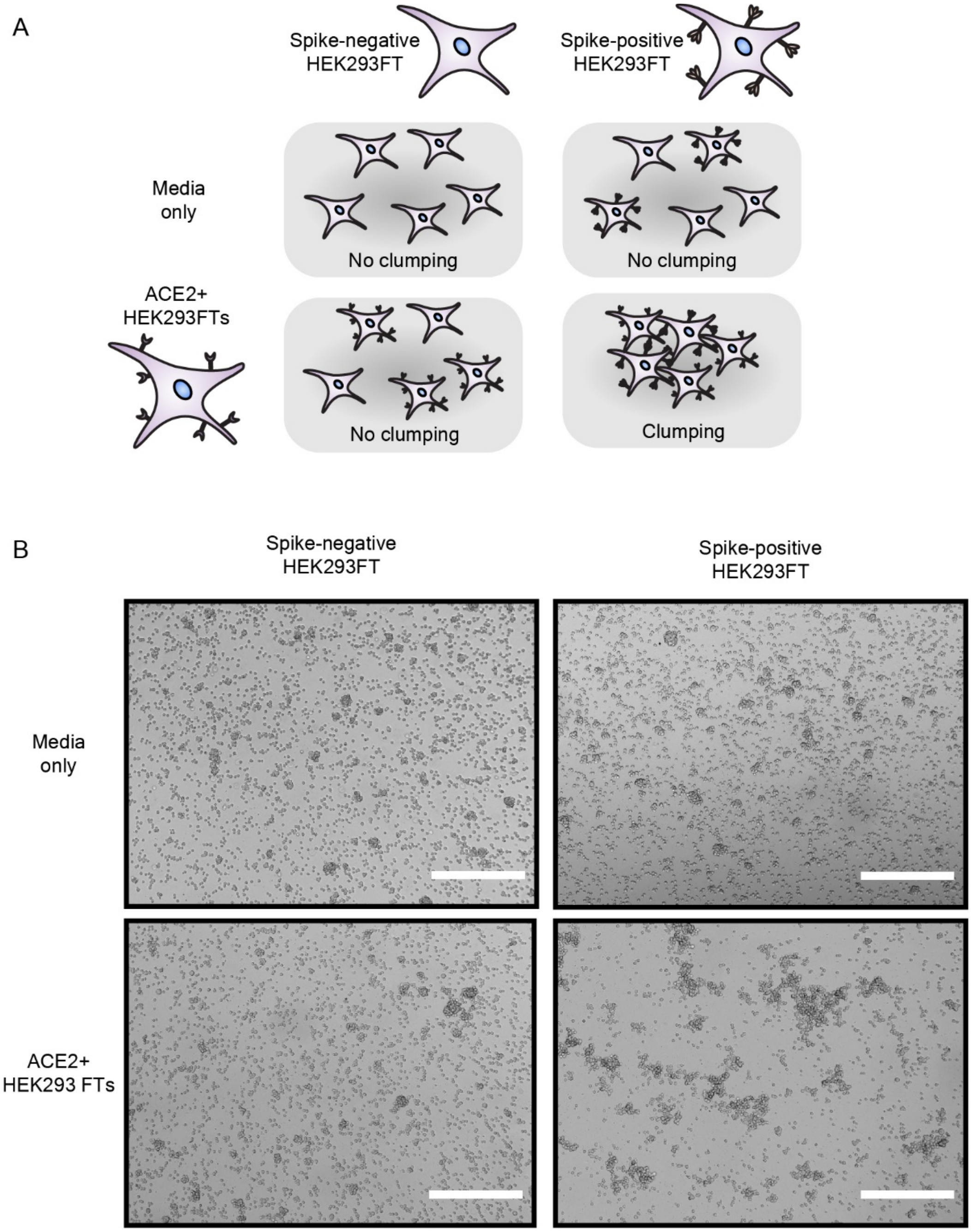

Figure S2. Cells expressing Spike and ACE2 bind and clump. A) Cartoon depicting the cell binding assay. Cells expressing ACE2 or a media-only control sample are mixed with freshly plated Spike-positive or Spike-negative cells such that the total number of cells is equal for all conditions. Spike-positive and Spike-negative cells are a HEK293FT cell line engineered to inducibly express the Spike protein in the presence of doxycycline. Spike-positive cells in this assay have been cultured with doxycycline for greater than 24 h prior to assay, while Spike-

negative cells are not cultured in doxycycline. If the binding interaction is specific, clumping is only expected for conditions where both ACE2 and Spike are present in trans. B) Representative microscopy images demonstrating cell binding as depicted in A (n = 2). Scale bar is 500 μm.

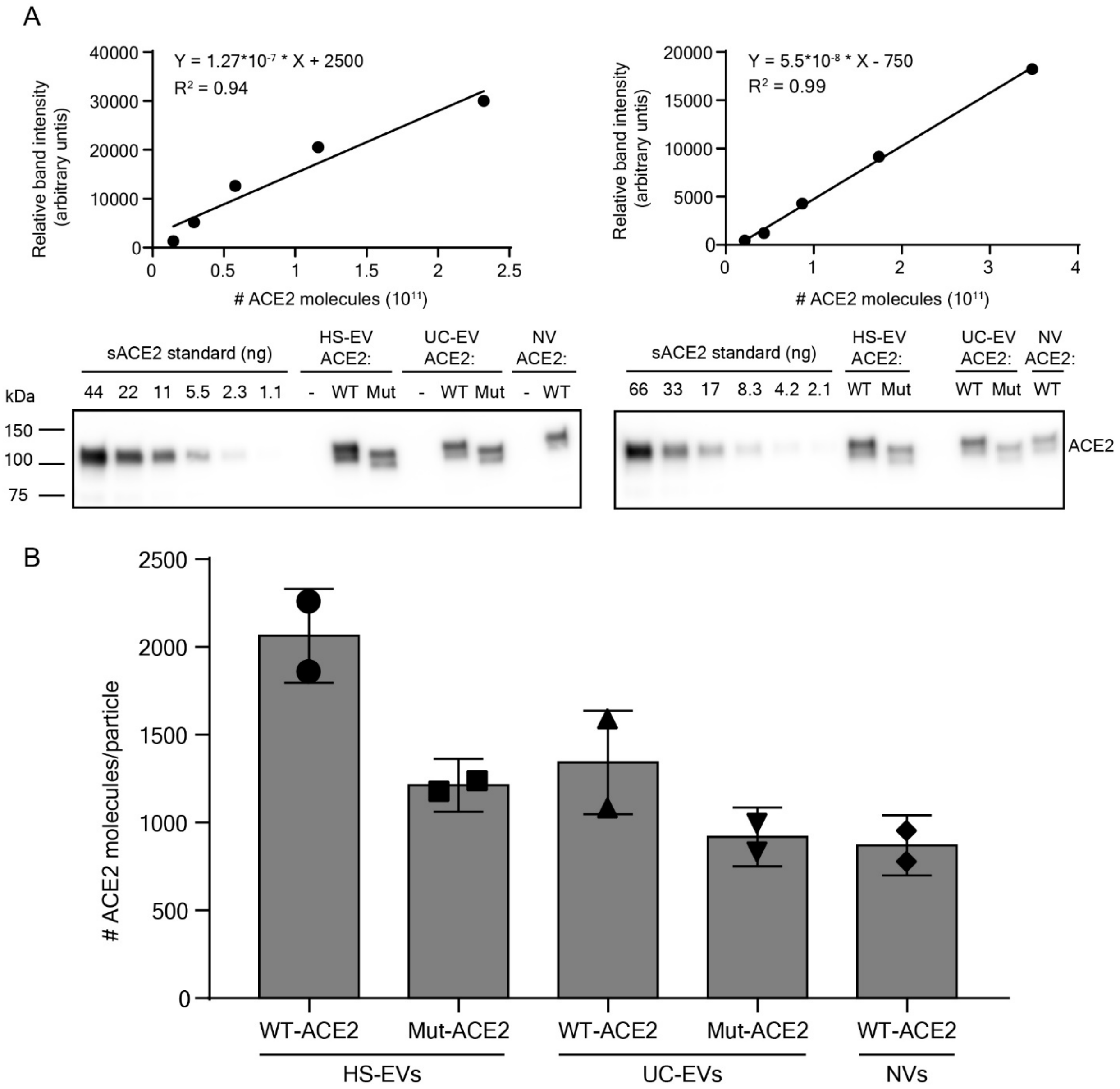

Figure S3. Semiquantitative western blots of the ACE2 content of vesicles. A) sACE2 was run on a western blot against an equal number of different vesicle subtypes using an anti-ACE2 primary antibody (MAB933). Calibration curves were generated from the intensity of sACE2 bands on the western blots; the greatest dilution in each blot was excluded from this analysis because of low signal. Equations for each curve are shown. Two independent replicates of this experiment were completed. B) ACE2 quantity per vesicle using the calibration curves derived in A. Symbols represent a single value calculated from a single calibration curve; bar graphs represent the mean of the two individually calculated values. Error bars are the square root of the sum of the variances

due to the standard error of the mean between the two data points and the propagated error from the individual measurements.

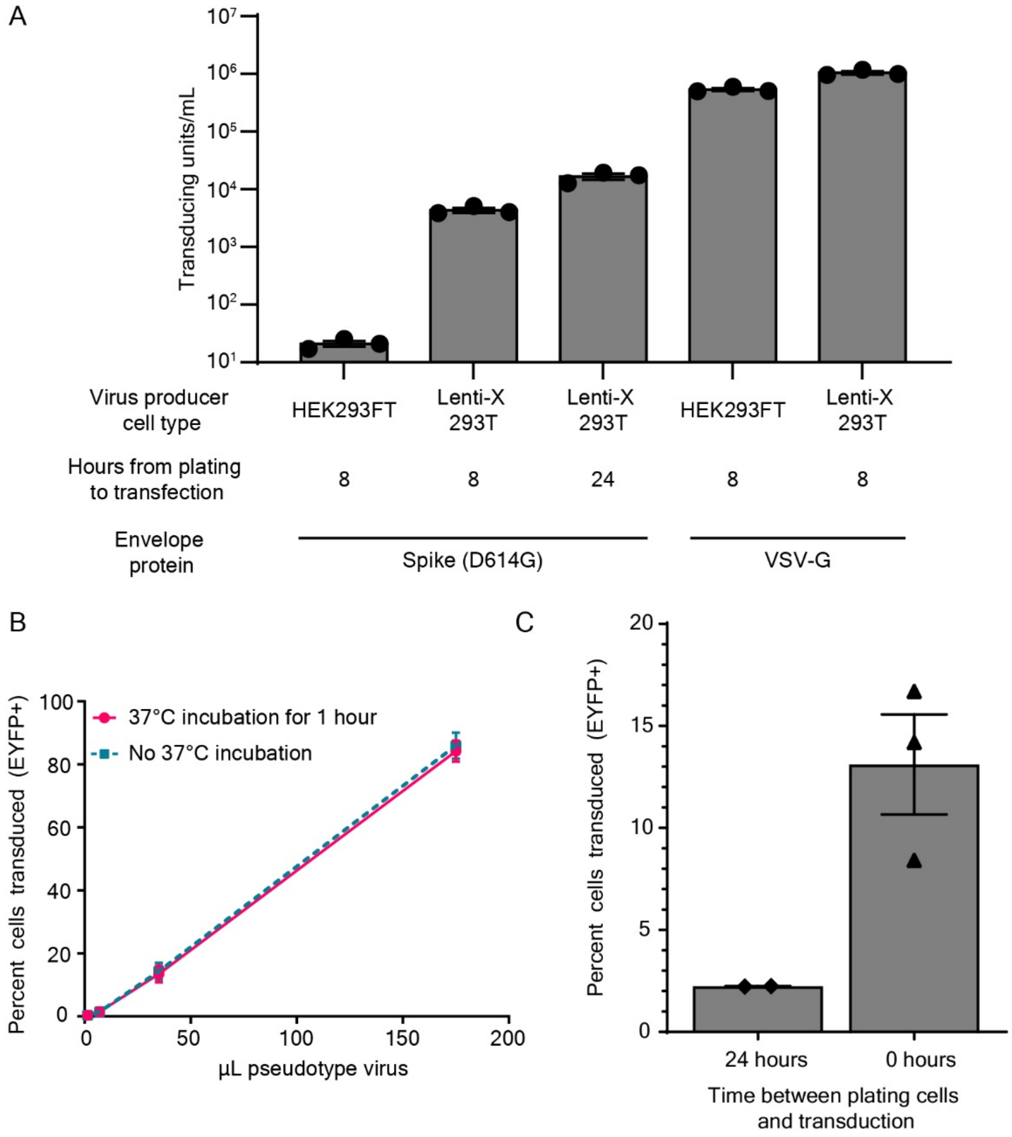

Figure S4. Viral producer cell type and transduction conditions affect SARS-CoV-2 Spike-lenti titer. A) Functional viral titer for Spike-lenti and VSV-G pseudotyped lentivirus generated from HEK293FT and HEK293T Lenti-X cells. Viral producing cells were plated at the time indicated before transfection of viral plasmids. Virus was titered on WT-ACE2-expressing HEK293FTs, and titer was determined using flow cytometry. Titer is presented as transducing units (TU) / mL of unconcentrated viral preparation. Each symbol represents a biological replicate, the bar graph represents the mean of those replicates, and the error bars represent the standard error of the mean. One independent experiment was performed. B) Percent of cells transduced with different amounts

of Spike-lenti that was either exposed to 37°C for 1 h or left on ice prior to transduction. Symbols represent the average from three biological replicates; error bars represent standard deviation. One independent experiment was performed. C) Percent of cells transduced for Spike-lenti for target cells plated at different times prior to inoculation. 4 x 10^3 target cells were plated in both conditions and all conditions received the same volume of virus. ACE2-expressing HEK293FTs were more susceptible to transduction when plated at the time of transduction, effectively increasing viral titer. Spike-lenti in C) was harvested from HEK293FTs at 44 h after the media change; this virus not incubated for 1 h at 37°C or centrifuged at 500 g for 1 min prior to inoculation. Media was not changed after 16 h as in other experiments. Each symbol represents a biological replicate, the bar graph represents the mean of those replicates, and the error bars represent the standard error of the mean. One independent experiment was performed.

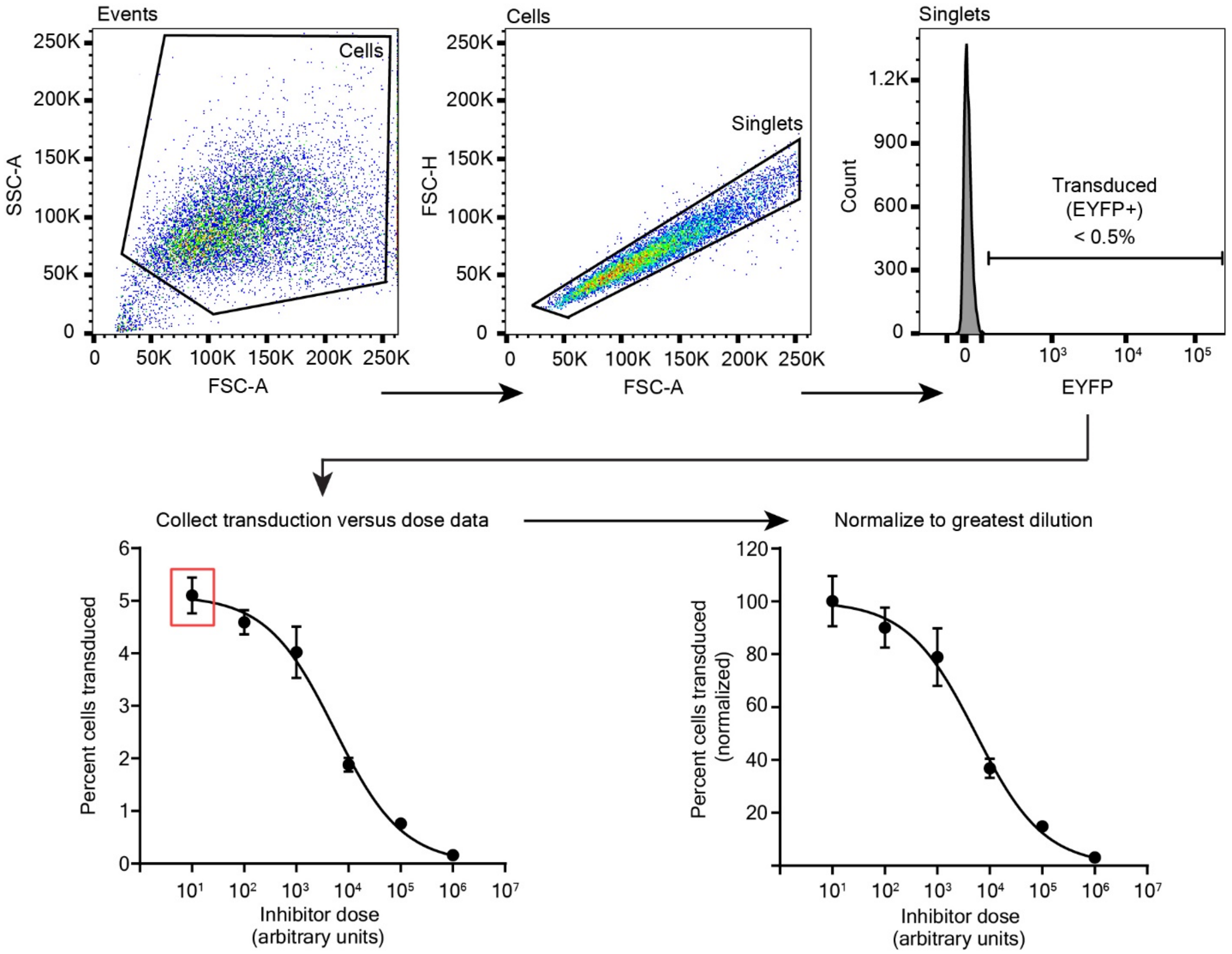

Figure S5. Data analysis workflow for viral inhibition experiments. First, events recorded via analytical flow cytometry are gated for cells (via side scatter-area (SSC-A) vs forward scatter-area (FSC-A) discrimination) and then for single cells (via forward scatter-height (FSC-H) vs FSC-A discrimination). Transduced cells are then determined as cells that have higher EYFP fluorescence than 99.5% of single cells in a population not exposed to virus. Percent of cells transduced, as determined by the aforementioned gating, are then plotted against inhibitor dose. Percent cells transduced is then normalized to the percent transduction in the greatest dilution in a given treatment's series, here indicated with a red square.

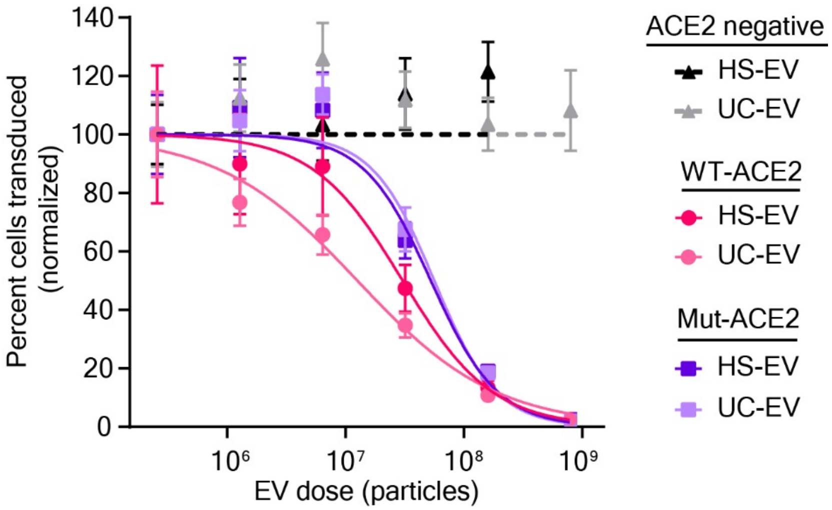

Figure S6. Decoy EVs inhibit Spike-lenti. These data represent an independent replicate of the experiment reported in **Figure 3D**. Dose-response curve demonstrating the relationship between decoy vesicle dose and normalized percentage of cells transduced by Spike-lenti. Curves are normalized to the percent of cells transduced at the lowest vesicle dose in a particular curve. Symbols represent the mean of three biological replicates, and the error bars are standard error of the mean.

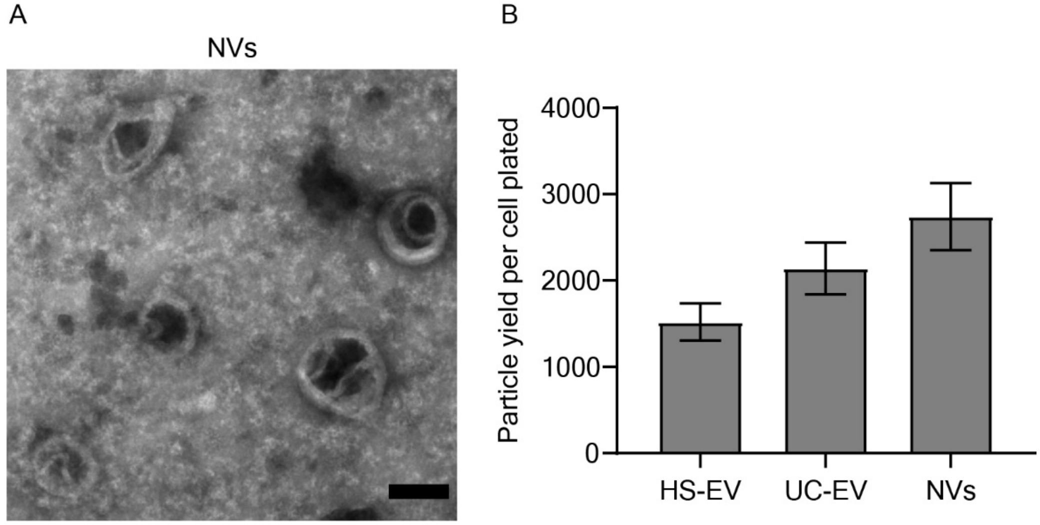

Figure S7. NVs display unique morphology and provide similar particle yields to EVs. A) Transmission electron microscopy images depicting the presence of suspected multilamellar vesicles in a NV sample. Scale bar is 100 nm. B) Particle yield for NVs versus HS-EVs and UC-EVs normalized to the initial number of wild-type ACE2-engineered cells plated for the preparation. Error bars represent the standard deviation of a single vesicle preparation based on propagated error.

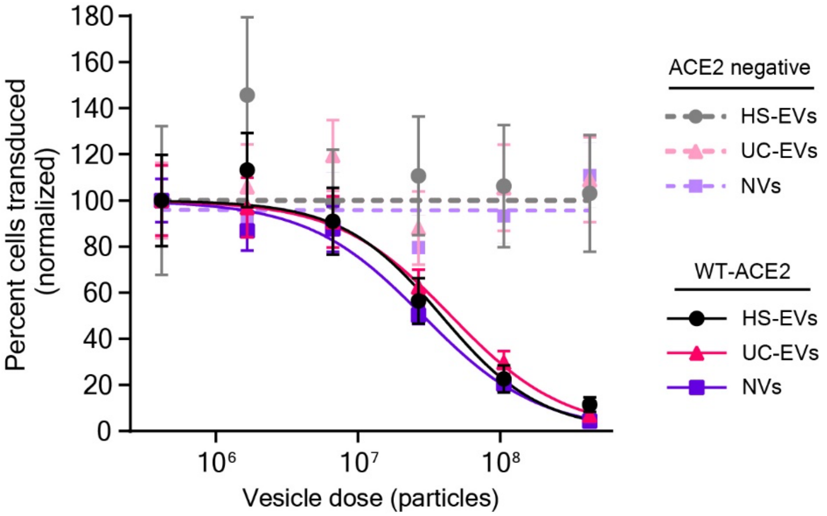

Figure S8. ACE2 NVs inhibit Spike-lenti. These data are an independent replicate of the experiment reported in **Figure 4F**. Dose-response curve demonstrating the relationship between decoy vesicle dose and normalized percentage of cells transduced by Spike-lenti. Curves are normalized to the percent of cells transduced at the lowest vesicle dose in a particular curve. Symbols represent the mean of three biological replicates except for WT-ACE2 HS-EVs, where the third dilution in the series (2.7 x 10^7 particles) is the mean of two biological replicates. Error bars are standard error of the mean.

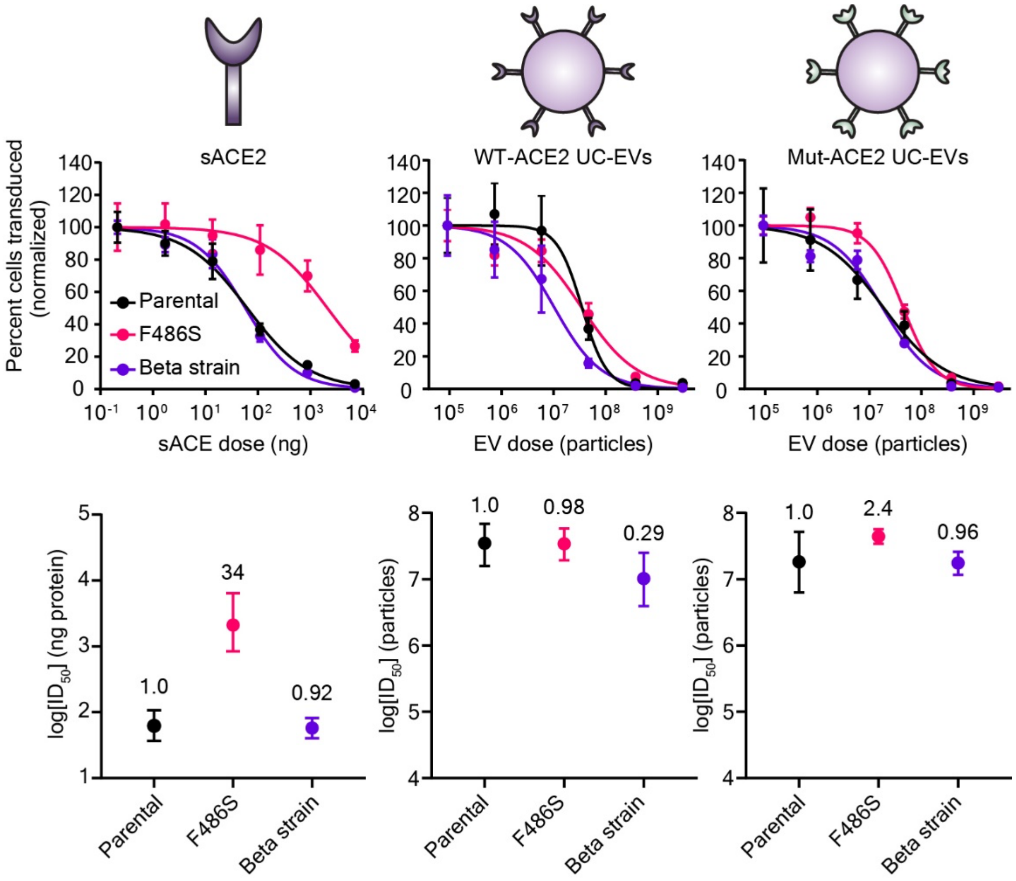

Figure S9. Decoy vesicles maintain inhibition potency against Spike-lenti mutants. These data represent an independent replicate of the experiment reported in **Figure 5B**. Top: Dose-response curves depicting the inhibition of various strains of SARS-CoV-2 Spike-lenti by sACE2, WT-ACE2 UC-EVs, and Mut-ACE2 UC-EVs. Curves are normalized to the percent of cells transduced at the lowest vesicle dose in a particular curve. Symbols represent the mean of three biological replicates, and the error bars are standard error of the mean. Bottom: Log(ID_{50}) values calculated from the data in the top portion of this panel. Numbers above each point report the relative resistance for a given strain relative to the parental (D614G) strain for that particular inhibitor treatment. Error bars represent 95% confidence intervals for the parameter (ID_{50}) estimation.

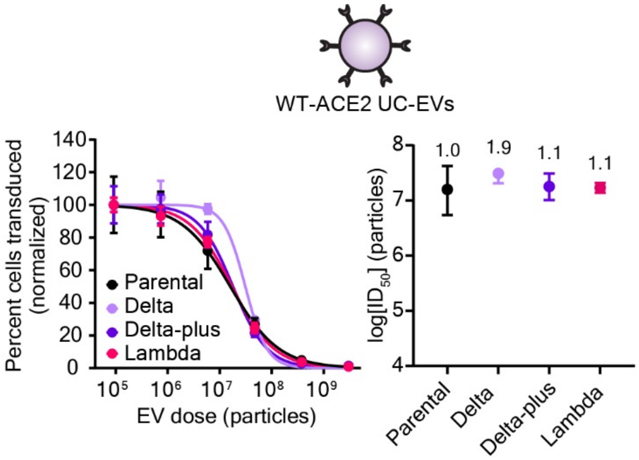

Figure S10. Decoy vesicles maintain inhibition potency against emerging Spike-lenti mutants. These data represent an independent replicate of the experiment reported in **Figure 6**. Left: Dose-response curves depicting the inhibition of various strains of SARS-CoV-2 Spike-lenti by WT-ACE2 UC-EVs. The parental strain is the D614G Spike-lenti. Curves are normalized to the percent of cells transduced at the lowest EV dose in a particular curve. Symbols represent the mean of three biological replicates except for the parental strain, where the fourth dilution in the series (5.9 x 10^6 particles) represents two biological replicates. Error bars are standard error of the mean. Right: Log(ID_{50}) values calculated from the dose response curves. Numbers above each point report the relative resistance for a given strain relative to the parental strain (D614G). Error bars represent 99% confidence intervals for the parameter (ID_{50}) estimation. No upper confidence interval is reported for the delta condition because the analysis method used did not provide a reliable upper bound for this parameter.

Table S1. Calculated ID_{50} and IC_{50} parameters and errors for viral inhibition experiments investigating the effect of ACE2 mutant and EV subtype on Spike-lenti transduction corresponding to **Figure 3D**.

	ID_{50} [x 10^7 Particles]		IC_{50} [x 10^7 Particles]	
Sample	Best Fit	Uncertainty (95% C.I.) [-,+]	Best Fit	Uncertainty (95% C.I.) [-,+]
WT-ACE2 HS-EV	2.8	[0.7, 1]	14	[4, 5]
WT-ACE2 UC-EV	3.8	[0.9, 1]	19	[4, 6]
Mut-ACE2 HS-EV	1.3	[0.5, 0.7]	6.5	[2, 3]
Mut-ACE2 UC-EV	2.2	[0.4, 0.5]	11	[2, 2]

Table S2. Calculated ID_{50} and IC_{50} parameters and errors for viral inhibition experiments investigating the effect of ACE2 mutant and EV subtype on Spike-lenti transduction corresponding to **Figure S6**.

	ID_{50} [x 10^7 Particles]		IC_{50} [x 10^7 Particles]	
Sample	Best Fit	Uncertainty (95% C.I.) [-,+]	Best Fit	Uncertainty (95% C.I.) [-,+]
WT-ACE2 HS-EV	3.0	[2, 4]	15	[8, 20]
WT-ACE2 UC-EV	1.3	[0.5, 0.9]	6.3	[3, 5]
Mut-ACE2 HS-EV	5.3	[2, 4]	26	[10, 20]
Mut-ACE2 UC-EV	5.6	[2, 3]	28	[9, 10]

Table S3. Calculated ID_{50} and IC_{50} parameters and errors for viral inhibition experiments investigating the potency of WT-ACE2 EVs versus cell-derived NVs on Spike-lenti transduction corresponding to **Figure 4F**.

	ID_{50} [x 10^7 Particles]		ID_{50} [x 10^7 Particles]	
Sample	Best Fit	Uncertainty (95% C.I.) [-,+]	Best Fit	Uncertainty (95% C.I.) [-,+]
HS-EVs	2.3	[0.7, 1]	11	[3, 5]
UC-EVs	3.6	[1, 2]	18	[7, 10]
NV	2.0	[0.6, 0.8]	10.	[3, 4]

Table S4. Calculated ID_{50} and IC_{50} parameters and errors for viral inhibition experiments investigating the potency of WT-ACE2 EVs versus cell-derived NVs on Spike-lenti transduction corresponding to **Figure S8**.

	ID_{50} [x 10^7 Particles]		ID_{50} [x 10^7 Particles]	
Sample	Best Fit	Uncertainty (95% C.I.) [-,+]	Best Fit	Uncertainty (95% C.I.) [-,+]
HS-EVs	3.9	[2, 4]	19	[10, 20]
UC-EVs	4.5	[2, 3]	22	[9, 20]
NV	2.9	[0.9, 1]	14	[5, 7]

Table S5. Calculated ID_{50}, IC_{50}, ID_{50} TU^{-1}, and relative resistance parameters for viral inhibition experiments investigating the potency of UC-EVs and sACE2 against Spike-lenti mutants corresponding to **Figure 5B**.

Inhibitor	Virus	ID_{50} [x 10^7 particles or ng protein]	Relative resistance	IC_{50} [x 10^7 particles mL^{-1} or ng protein mL^{-1}]	ID_{50} TU^{-1} [particles TU^{-1} or ng protein TU^{-1}]	Relative resistance-TU normalized
sACE2	Parental	83	1.0	420	0.55	1.0
	sACE2 Resistant	4400	53	22000	46	84
	Beta Strain RBD	43	0.51	210	0.18	0.34
WT-ACE2 UC-EVs	Parental	2.0	1.0	9.9	200000	1.0
	sACE2 Resistant	1.5	0.76	7.5	130000	0.66
	Beta Strain RBD	0.32	0.16	1.6	13000	0.066
Mut-ACE2 UC-EVs	Parental	1.7	1.0	8.6	120000	1.0
	sACE2 Resistant	1.6	0.93	8.0	140000	1.2
	Beta Strain RBD	1.1	0.62	5.3	52000	0.42

Table S6. Calculated ID_{50}, IC_{50}, and ID_{50} TU^{-1} parameters converted to numbers of molecules of ACE2 for viral inhibition experiments investigating the potency of UC-EVs and sACE2 against Spike-lenti mutants corresponding to **Figure 5B**.

Inhibitor	Virus	ID_{50} [x 10^9 molecules]	IC_{50} [x 10^9 molecules mL^{-1}]	ID_{50} TU^{-1} [x 10^9 molecules TU^{-1}]
sACE2	Parental	440	2200	2.9
	sACE2 Resistant	23000	120000	240
	Beta Strain RBD	220	1100	0.96
WT-ACE2 UC-EVs	Parental	27	130	0.26
	sACE2 Resistant	20	100	0.17
	Beta Strain RBD	4.3	22	0.017
Mut-ACE2 UC-EVs	Parental	16	79	0.11
	sACE2 Resistant	15	73	0.13
	Beta Strain RBD	9.8	49	0.048

Table S7. Calculated ID_{50}, IC_{50}, ID_{50} TU^{-1}, and relative resistance parameters for viral inhibition experiments investigating the potency of UC-EVs and sACE2 against Spike-lenti mutants corresponding to **Figure S9**.

Inhibitor	Virus	ID_{50} [x 10^7 particles or ng protein]	Relative resistance	IC_{50} [x 10^7 particles mL^{-1} or ng protein mL^{-1}]	ID_{50} TU^{-1} [particles TU^{-1} or ng protein TU^{-1}]	Relative resistance-TU normalized
sACE2	Parental	62	1.0	310	0.30	1.0
	sACE2 Resistant	2100	34	11000	15	50
	Beta Strain RBD	58	0.92	290	0.42	1.4
WT-ACE2 UC-EVs	Parental	3.5	1.0	18	290000	1.0
	sACE2 Resistant	3.4	0.98	17	230000	0.79
	Beta Strain RBD	1.0	0.29	5.1	66000	0.23
Mut-ACE2 UC-EVs	Parental	1.8	1.0	9.2	120000	1.0
	sACE2 Resistant	4.4	2.4	22	340000	2.9
	Beta Strain RBD	1.8	0.96	8.8	150000	1.3

Table S8. Calculated ID_{50}, IC_{50}, and ID_{50} TU^{-1} parameters converted to numbers of molecules of ACE2 for viral inhibition experiments investigating the potency of UC-EVs and sACE2 against Spike-lenti mutants corresponding to **Figure S9**.

Inhibitor	Virus	ID_{50} [x 10^9 molecules]	IC_{50} [x 10^9 molecules mL^{-1}]	ID_{50} TU^{-1} [x 10^9 molecules TU^{-1}]
sACE2	Parental	330	1600	1.6
	sACE2 Resistant	11000	55000	78
	Beta Strain RBD	300	1500	2.2
WT-ACE2 UC-EVs	Parental	47	230	0.38
	sACE2 Resistant	46	230	0.30
	Beta Strain RBD	14	69	0.088
Mut-ACE2 UC-EVs	Parental	17	84	0.11
	sACE2 Resistant	41	200	0.31
	Beta Strain RBD	16	81	0.14

Table S9. Calculated ID_{50}, IC_{50}, ID_{50} TU^{-1}, and relative resistance parameters for viral inhibition experiments investigating the potency of WT-ACE2 UC-EVs against Spike-lenti mutants corresponding to **Figure 6**.

Virus	ID_{50} [x 10^7 particles]	Relative resistance	IC_{50} [x 10^7 particles mL^{-1}]	ID_{50} TU^{-1} [particles TU^{-1}]	Relative resistance-TU normalized
Parental	3.1	1.0	15	81000	1.0
Delta	2.1	0.70	11	79000	0.98
Delta-plus	1.4	0.47	7.1	70000	0.87
Lambda	2.4	0.79	12	87000	1.1

Table S10. Calculated ID_{50}, IC_{50}, ID_{50} TU^{-1}, and relative resistance parameters for viral inhibition experiments investigating the potency of WT-ACE2 UC-EVs against Spike-lenti mutants corresponding to **Figure S10**.

Virus	ID_{50} [x 10^7 particles]	Relative resistance	IC_{50} [x 10^7 particles mL^{-1}]	ID_{50} TU^{-1} [particles TU^{-1}]	Relative resistance-TU normalized
Parental	1.6	1.0	8.0	24000	1.0
Delta	3.1	1.9	16	46000	1.9
Delta-plus	1.8	1.1	9.0	31000	1.3
Lambda	1.7	1.1	8.5	24000	1.0

Table S11. Western blot antibodies used in this study and associated sample preparation considerations.

Antibody target	Supplier (#)	Denature temperature / time	Antibody dilution	Reducing/Non-reducing Laemmli	Animal of origin
HA-tag	Cell Signaling Technology (C29F4)	70°C / 10 min	1:1000	Reducing	Rabbit
ACE2 (C-term)	Abcam (Ab15348)	70°C / 10 min	1:1000	Reducing	Rabbit
ACE2 (N-term)[a)]	R&D Systems (MAB933)	95°C / 5 min	1:1000	Reducing	Mouse
CD9	Santa Cruz (sc-13118)	95°C / 10 min	1:500	Reducing	Mouse
CD81	Santa Cruz (sc-23962)	95°C / 10 min	1:500	Non-reducing	Mouse
Alix	Abcam (Ab117600)	95°C / 10 min	1:500	Reducing	Mouse
Calnexin	Abcam (Ab22595)	70°C / 10 min	1:1000	Reducing	Rabbit
Rabbit	Invitrogen (32460)	Not applicable	1:3000	Not applicable	Goat
Mouse	Cell Signaling Technology (7076)	Not applicable	1:3000	Not applicable	Horse

[a)] Resuspended in PBS at recommended concentration (0.5 mg/mL)
